# Biomimetic Dexterous Hand Control for Robotic Piano Playing Using a Two-Stage Reinforcement Learning Curriculum

**DOI:** 10.3390/biomimetics11090610

**Published:** 2026-08-28

**Authors:** Lei Jiang, Jinyi Chen, Kaixin Lan, Xianwei Liu, Yongbin Jin, Hongtao Wang

**Affiliations:** 1 Center for X-Mechanics, Zhejiang University, Hangzhou 310012, China; 12524001@zju.edu.cn (L.J.); cjyy@zju.edu.cn (J.C.); 22424122@zju.edu.cn (K.L.); 3140104175@zju.edu.cn (X.L.); 2 State Key Laboratory of Fluid Power and Mechatronic System, Zhejiang University, Hangzhou 310012, China; 3 ZJU-Hangzhou Global Scientific and Technological Innovation Center, Zhejiang University, Hangzhou 311200, China

**Keywords:** dexterous hand, robotic piano playing, reinforcement learning, curriculum learning, sim-to-real transfer

## Abstract

Robotic piano playing is a challenging benchmark for biomimetic dexterous manipulation, requiring precise timing, coordinated multi-finger motion, and stable key contact. This study proposes a robotic piano-playing framework based on a two-stage reinforcement learning curriculum. Musical Instrument Digital Interface (MIDI) data are converted into target-key and fingering grids to provide future musical goals for policy learning in a parallel MJLab simulation environment. A Soft Actor–Critic (SAC) agent takes a 2106-dimensional observation vector, including joint states, previous actions, musical phase, future key targets, fingering assignments, and piano-key states, and outputs a 21-dimensional continuous action vector for wrist, finger, and global hand-positioning control. Stage 1 weakens physical regularization to facilitate key-pressing acquisition, whereas Stage 2 strengthens power, velocity, acceleration, collision, posture, and finger-speed constraints to improve the regularity of policy outputs and readiness for real-world deployment. Simulation experiments on 30 s right-hand excerpts from Für Elise, Canon, and Beethoven’s Symphony No. 5 achieve frame-wise key-state F1 scores above 0.99 on the first two excerpts and approximately 0.945 on Beethoven. Real-world deployment uses open-loop playback of policy-generated high-level trajectories with low-level joint-position feedback and achieves F1 scores of 0.95, 0.91, and 0.83, respectively, while reproducing representative piano techniques such as chords, octaves, mixed black-and-white-key patterns, overlapping finger actions, and rapid sequential movements. These physical results demonstrate the feasibility of the proposed sim-to-real pipeline for complete 30 s executions; they are not intended as a statistical repeatability study. The results further show that biomimetic robotic hands can learn complex piano-playing skills from MIDI-based task objectives without relying on human motion demonstration trajectories.

## 1. Introduction

Human hands exhibit remarkable dexterity in musical performance [1,2,3]. During piano playing, the fingers must independently and cooperatively execute rapid key presses, maintain accurate timing, avoid unintended contacts, and continuously reshape the hand to accommodate changing musical structures [1,2]. These abilities are not limited to simple point-to-point motion; rather, they reflect an integrated form of biomimetic manipulation involving multi-finger coordination, contact regulation, timing prediction, and posture adaptation [4]. From a biomechanical perspective, human pianists rely on anticipatory hand shaping—adjusting finger spacing, wrist orientation, and forearm position before key contact—to facilitate accurate and efficient key pressing [1,2]. The neural control of intrinsic and extrinsic hand muscles enables both independent finger movements and synergistic coordination, a principle that has been extensively studied in single-finger pressing tasks [5] and is directly relevant to multi-finger piano performance. Therefore, robotic piano playing provides a challenging and meaningful task for evaluating biomimetic robotic hands, as it demands not only dexterous actuation but also biologically inspired control strategies that emulate human preparatory and coordinative mechanisms.

Compared with conventional robotic manipulation tasks such as reaching, grasping, or button pressing, piano playing imposes stricter requirements on both spatial and temporal precision. A robotic pianist must press the correct key at the correct time while avoiding neighboring keys. In addition, complex musical passages may include double notes, three-finger chords, octaves, overlapping finger actions, mixed black-and-white-key chords, black-and-white double notes, repeated notes, rapid sequential patterns, and chromatic repetitions. These techniques require coordinated finger selection, anticipatory wrist and hand posture control, and stable contact between fingertips and piano keys [1,6,7]. Historically, keyboard-playing robots such as WABOT-2 and the ACT Hand have also shown that piano performance can expose the coupled demands of hand morphology, finger-arm coordination, and timing control [6,8]. As a result, piano playing is particularly suitable for testing biomimetically inspired multi-finger manipulation skills in a structured yet demanding environment.

Recent studies in robot learning have demonstrated the potential of deep reinforcement learning, robot learning hardware, and imitation learning for high-dimensional dexterous control [9,10,11,12,13,14,15,16,17]. However, applying these methods to robotic piano playing remains challenging. First, the action space of a dexterous robotic hand is high dimensional, and the contact dynamics between fingertips and piano keys are sensitive to small errors [12,13,14,16]. Second, the reward is highly time-dependent: a note is successful only when its pitch and onset timing match the target musical sequence. Third, policies trained in simulation may produce excessive joint velocity, abrupt action changes, or unstable contact, which can hinder physical deployment [14,18,19,20,21,22]. Fourth, many existing approaches rely on human demonstrations, captured hand-motion data, or internet performance videos, which can be expensive to collect and difficult to generalize across musical pieces and robotic embodiments [10,17,23,24].

To address these challenges, we draw inspiration from three key principles of human piano biomechanics: (i) anticipatory hand shaping—pianists prepare finger positions and wrist orientation before note onset, which we implement through a 21-step lookahead observation window; (ii) finger-to-key assignment—the natural mapping between fingers and keys reduces motion ambiguity, which we encode explicitly via a fingering grid in the observation and reward; and (iii) progressive skill acquisition—human pianists first learn note accuracy and later refine motion efficiency and expressiveness, which we emulate through a two-stage curriculum separating key-pressing acquisition from motion refinement.

**Figure 1:**
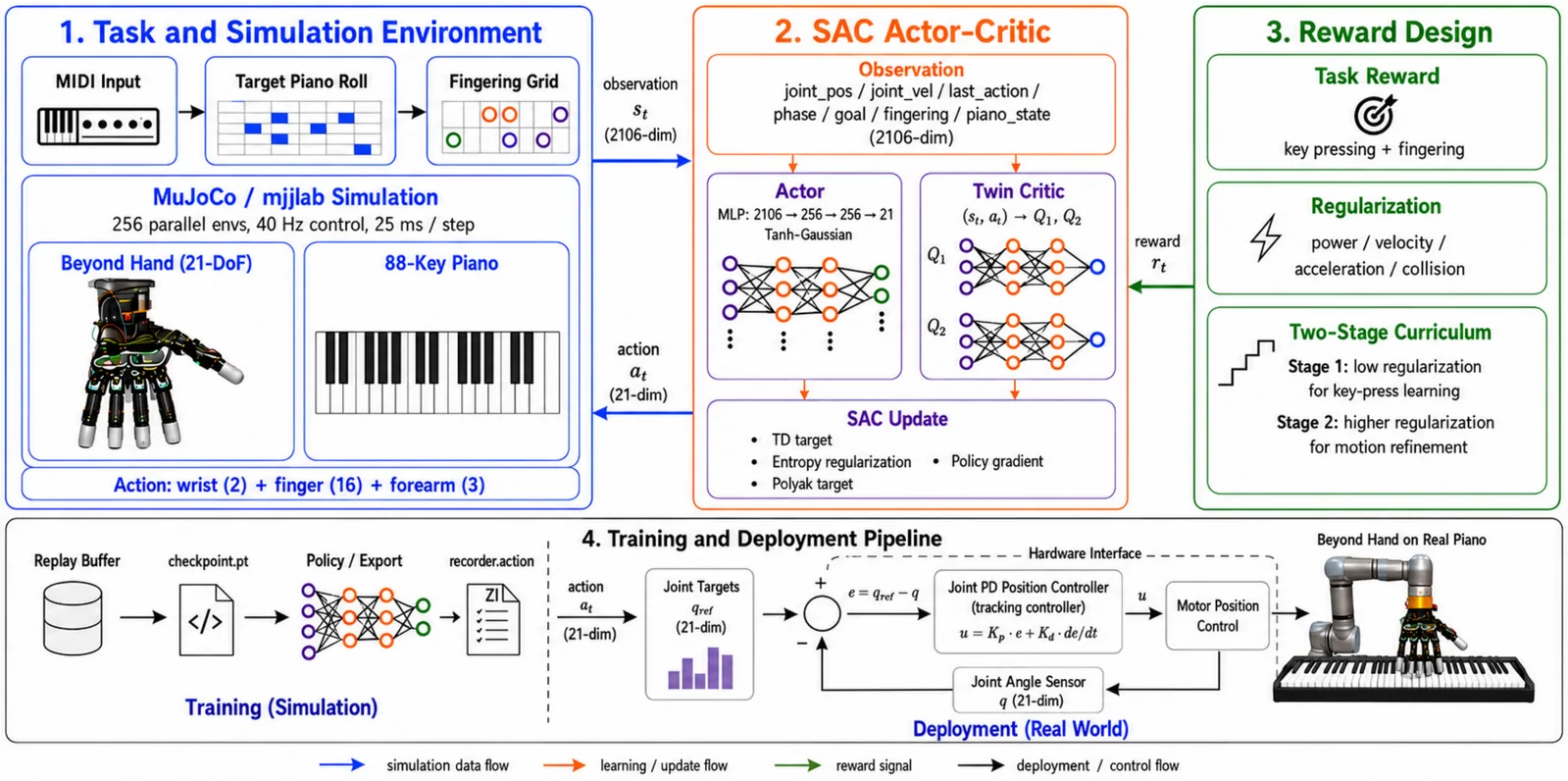
Overall framework of the proposed biomimetic robotic piano-playing system. The framework integrates MIDI-based task encoding, SAC-based policy training, two-stage curriculum learning, and sim-to-real deployment with PD-based joint position control.

Building on these biomimetic principles, this paper presents a robotic piano-playing framework based on a two-stage reinforcement learning curriculum [25,26,27]. The framework builds upon and extends the RoboPianist environment [12] in several key aspects: while RoboPianist demonstrated the feasibility of RL for piano playing using a generic observation and reward design, we introduce (i) an observation space that explicitly encodes future musical targets and fingering assignments for anticipatory control; (ii) a two-stage curriculum that separates key-press acquisition from motion refinement; (iii) a reward formulation with stage-dependent physical regularization, including finger-speed and posture constraints; and (iv) a sim-to-real deployment pipeline on a different physical robotic platform. The overall system is shown in Figure 1. The framework takes MIDI data as input, converts it into target piano-key and fingering grids, trains a robotic hand in a parallel MJLab/MuJoCo environment [28,29], and deploys the learned action trajectories to a physical Beyond Hand robotic system [30]. The core learning algorithm is Soft Actor–Critic (SAC) [31,32]. The actual SAC implementation and optimization parameters used in the reported runs are specified in Section 2.7 rather than being treated as independently validated algorithmic contributions.

The main contributions of this work are as follows:

1. We introduce a structured MIDI-to-control representation that jointly encodes future key targets, fingering assignments, musical phase, robotic proprioception, and piano-key states, enabling anticipatory multi-finger control without requiring human motion trajectories.
2. We propose a two-stage reinforcement learning curriculum that separates key-pressing acquisition from motion refinement through stage-dependent task rewards and physical regularization, providing a practical route from simulation learning to hardware-ready trajectory generation.
3. We validate the learned policy-generated trajectories on a physical Beyond Hand piano-playing platform using frame-wise MIDI key-state evaluation and joint-angle tracking, demonstrating sim-to-real feasibility for representative complex piano-playing techniques.

The remainder of this paper is organized as follows. Section 2 describes the materials and methods, including the robotic piano-playing platform, MIDI-based task representation, parallel MJLab simulation environment, observation and action spaces, Soft Actor–Critic policy learning, two-stage curriculum, reward formulation, evaluation metrics, and sim-to-real deployment pipeline. Section 3 presents the experimental results, including the comparison with the original RoboPianist baseline, simulation performance on three representative piano excerpts, motion-stability analysis, learned piano techniques, ablation studies, real-world piano performance, pitch-time MIDI visualization, and joint-angle tracking results. Section 4 discusses the effectiveness of the proposed curriculum, the role of observation design and the reported SAC implementation, the sim-to-real performance, the implications for biomimetic dexterous manipulation, and the limitations of the current system. Section 5 concludes the paper.

## 2. Materials and Methods

### 2.1. Overview of the Proposed Framework

The proposed robotic piano-playing system consists of four main components: music and task input, simulation-based reinforcement learning, action and reward processing, and sim-to-real deployment. The overall control architecture is illustrated in Figure 1.

The left side of Figure 1 shows the music and task input module. MIDI data are first cropped into 30-s right-hand excerpts, followed by an additional 2-s silent tail. The musical information is then converted into two structured time-indexed representations: a target piano grid and a fingering grid. The target piano grid specifies which of the 88 piano keys should be pressed at each control step, while the fingering grid assigns a corresponding finger to each target key.

The central module represents the reinforcement learning agent. The policy is trained in an MJLab simulation environment using Soft Actor–Critic. The agent receives a 2106-dimensional observation vector and outputs a 21-dimensional continuous action vector. The actor network generates joint position commands, while two critic networks estimate the corresponding state-action values. The SAC update procedure includes critic learning, actor optimization, entropy tuning, and Polyak averaging for target-network updates.

The reward design is shown on the right side of Figure 1. The reward function consists of task-oriented terms, including key-pressing and fingering rewards, as well as regularization terms related to power consumption, velocity, acceleration, collisions, natural posture, and finger-speed constraints. These terms are organized through a two-stage curriculum: Stage 1 emphasizes accurate key pressing, whereas Stage 2 further improves the physical quality and naturalness of the motion [27].

The bottom part of Figure 1 illustrates the training and deployment pipeline. After training, the policy is evaluated using frame-wise key-state Precision, Recall, and F1 score (see Section 2.10 for the evaluation metrics). The generated action trajectory is then exported by a recorder and deployed to the physical robotic system. During real-world execution, the high-level action trajectory is played back open-loop, whereas the target joint positions are tracked by low-level position servos using joint-angle sensor feedback and PD control [20].

### 2.2. Biomimetic Robotic Piano-Playing Platform

**Figure 2:**
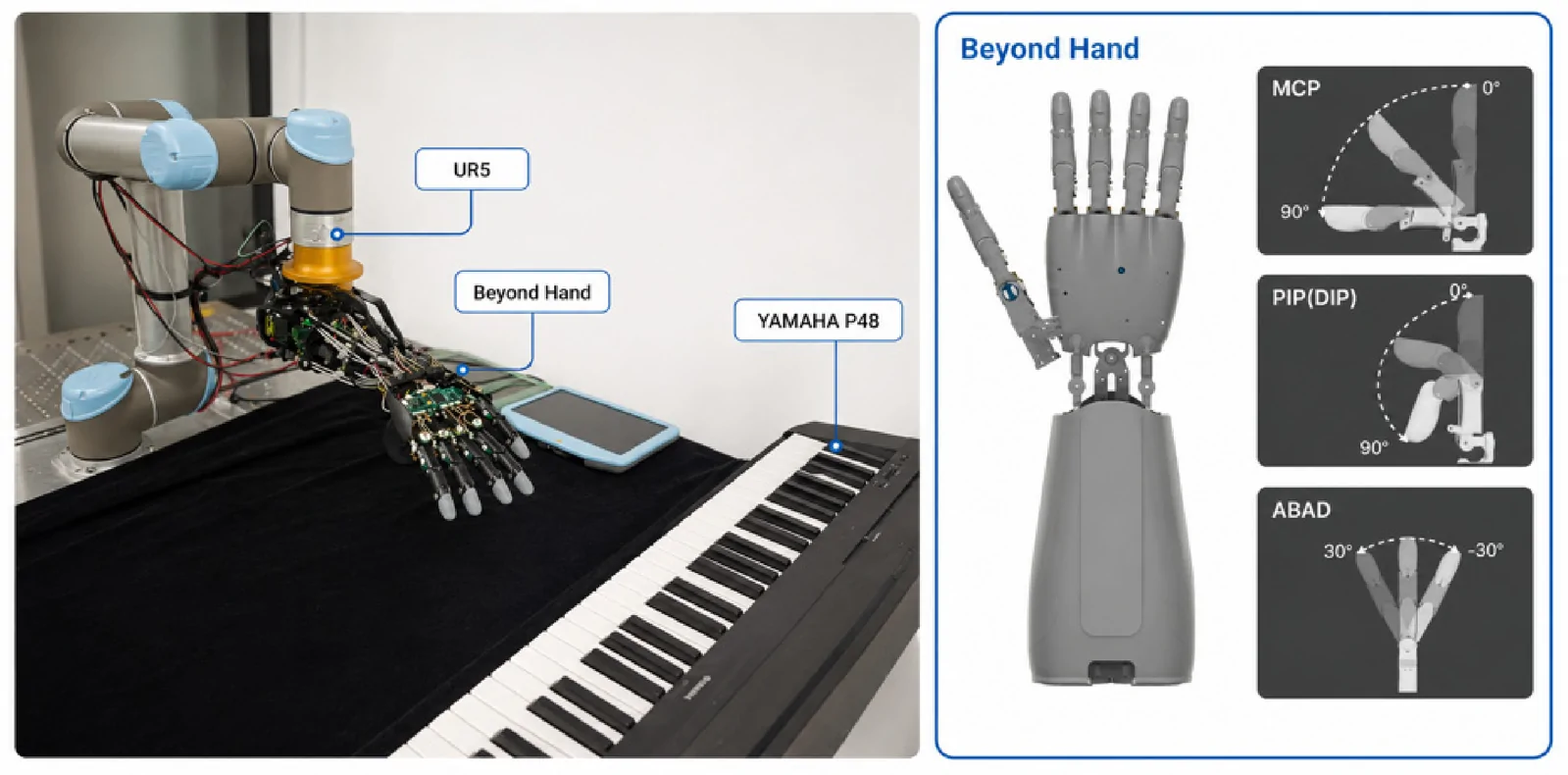
Physical robotic piano-playing platform. The platform consists of a UR5 robotic arm, a Beyond Hand dexterous hand, and a Yamaha digital piano.

The physical experimental platform is shown in Figure 2. It consists of a UR5 arm (Universal Robots, Odense, Denmark), a self-developed Beyond Hand (Zhejiang University, Hangzhou, China), and a Yamaha P-48B piano (Yamaha, Hamamatsu, Japan).

The Yamaha P-48B digital piano is used as the played instrument. It has an 88-key full-size keyboard and a graded hammer mechanism. The key weight gradually changes from the lower register to the higher register, providing a mechanical touch closer to that of an acoustic piano than a standard electronic keyboard. This property makes the instrument suitable for studying contact-rich robotic key pressing, since the robot must overcome realistic key resistance and key travel.

The piano is connected to the host computer through the USB TO HOST interface. This connection provides MIDI information, including note onset time, key number, and velocity. These signals are used to quantitatively evaluate the robot’s performance. The physical geometry of the keyboard is also important for robotic control. In the present setup, the white keys are approximately 22–23 mm wide, the black keys are approximately 10–13 mm wide, and the effective travel distance of a white key is approximately 9–11 mm. These measured geometric constraints directly influence fingertip design, contact modeling, and collision avoidance.

The UR5 robotic arm is used for global wrist positioning and large-scale movement across the keyboard. In piano performance, the arm is responsible for positioning the hand over the appropriate key region, while the dexterous hand performs local multi-finger key pressing. This division of labor is similar to human piano playing, where the arm and wrist provide global placement and the fingers execute local key actions.

The Beyond Hand robotic hand serves as the primary end-effector. It is designed with a biomimetic structure and multiple degrees of freedom, enabling anthropomorphic hand postures and coordinated finger movements [30]. In this work, the control action includes 21 dimensions: 2 wrist degrees of freedom, 16 finger degrees of freedom, and 3 forearm translational degrees of freedom. This configuration allows the system to learn not only individual finger presses but also coordinated wrist-finger-forearm behaviors, a control requirement that is also emphasized in recent anthropomorphic hand hardware and bimanual dexterous manipulation studies [15,16]. Table 1 summarizes the hardware, sensing, and control parameters directly relevant to the physical piano experiments.

In real-world execution, the high-level policy does not directly command motor torques. Instead, it outputs desired joint positions. These joint targets are sent to the hardware control layer, where each joint is controlled by a position servo. Joint-angle sensors provide real-time feedback, and the motor controller tracks the desired position using a PD control law [20]. This hierarchical design improves execution stability: the reinforcement learning policy focuses on generating coordinated target postures, while the low-level PD controller compensates for tracking errors and hardware disturbances [20].

### 2.3. Music Input and Task Encoding

The training tasks are derived from three representative right-hand piano excerpts: *Für Elise*, *Canon*, and *Beethoven’s Symphony No. 5*. These pieces were selected to cover different levels of musical difficulty. *Für Elise* contains relatively simple melodic structures, *Canon* includes denser finger transitions and repeated patterns, and the Beethoven excerpt contains more challenging fast movements and complex coordination.

Each excerpt is cropped to the first 30 s and followed by a 2 s silent tail. The silent tail ensures that the episode ends smoothly and allows the hand to release the keys after the musical segment. The original musical excerpts are read from MIDI files, while the fingering annotations are subsequently injected and maintained in the internal NoteSequence representation through the part field. This design allows time-aligned note and fingering information to be preserved during task encoding, since such fingering annotations are not reliably represented in standard MIDI files.

The score representation contains two main grids. The first is the target piano grid:

(1) $$G_{key}\in R^{T\times 88},$$

where T is the number of control steps and 88 corresponds to the full piano keyboard. Each element indicates whether a given key should be active at a given control step. The second is the fingering grid:

(2) $$G_{finger}\in R^{T\times 5},$$

where the five columns correspond to thumb, index, middle, ring, and little finger. This grid assigns target keys to individual fingers over time.

The musical phase is defined as

(3) $$\phi \left(t\right)=\frac{t}{T_{episode}},$$

where t is the current time index and $T_{episode}$ is the total episode length. To avoid discontinuity and provide a smooth temporal representation, the phase is encoded as $\left[cos(\pi \phi ),sin(\pi \phi )\right]$.

This phase encoding helps the policy distinguish different temporal locations within the same musical excerpt.

### 2.4. Parallel MJLab Simulation Environment

The reinforcement learning environment is implemented in MJLab. As shown in Figure 1, the simulated system contains a 21-degree-of-freedom robotic hand and an 88-key piano model. The proposed method uses 256 parallel simulation instances batched on a single GPU, thereby increasing data-collection throughput during training.

The physics timestep is 2.5 ms, corresponding to a simulation frequency of 400 Hz. The policy is executed every 10 physics steps, resulting in a control frequency of 40 Hz. Thus, each policy action is applied for 25 ms. This control frequency is suitable for piano-playing tasks because it provides sufficient temporal resolution for note onset control while keeping the learning problem computationally manageable.

A separate policy was trained for each 30 s musical excerpt. In the proposed implementation, one training step denotes one vectorized environment step: a single call to env.step(actions) advances all 256 parallel environments and nominally produces 256 individual environment transitions. Accordingly, checkpoint labels of 5000 and 20,000 vectorized steps correspond to approximately 1.28 million and 5.12 million individual transitions, respectively. After learning starts at vector step 1000, each outer step is followed by 32 SAC gradient iterations. By contrast, one step in the RoboPianist baseline denotes one single-environment interaction/transition. The raw step counts of the two implementations are therefore not directly comparable.

During training, the proposed method was evaluated every 1000 vectorized environment steps using a separate single-environment evaluator. The deterministic policy, without exploration noise, performed one complete rollout of the target musical excerpt at 40 Hz. Target and executed piano-key activation matrices were compared frame by frame to calculate frame-wise key-state Precision, Recall, and F1, and the mean values of the individual reward/penalty terms over the complete evaluation trajectory were also recorded. The RoboPianist baseline was evaluated every 100,000 single-environment steps using one deterministic full-episode rollout at 20 Hz.

Training was terminated when the predefined maximum number of environment steps was reached; no performance-based early-stopping criterion based on F1 score, critic loss, or reward convergence was used. For the formal repeated experiments, the proposed method used 20,000 vectorized environment steps for Stage 1 and a further 20,000 vectorized steps for Stage 2, while the RoboPianist baseline used 5,000,000 single-environment steps. Checkpoints were saved periodically, and retaining a best-performing checkpoint did not terminate training.

All reported training and evaluation were conducted locally in a Linux environment using a single NVIDIA GeForce RTX 4070 GPU with 12 GB of memory. The 256 proposed-method environments were batched on this same GPU rather than distributed across multiple GPUs, whereas the RoboPianist baseline used a single simulation environment. The exact CPU model, CPU utilization, system memory usage, and complete per-run wall-clock timing records were not reliably logged; therefore, we do not report a hardware-normalized wall-clock computational-efficiency comparison.

The piano model provides key-depth states for all 88 keys. Each key state is normalized to represent whether the key is released, partially pressed, or fully activated. The environment also provides fingertip-contact and hand-contact information, which is used to design collision and contact-related reward terms.

The environment outputs three main signals to the reinforcement learning agent: the observation $s_{t}$, the reward $r_{t}$, and the next-state transition after executing action $a_{t}$. The agent returns a 21-dimensional action vector, which is mapped to joint targets and applied to the simulated position actuators.

### 2.5. Observation Space

The observation vector is designed to combine robotic proprioception, action history, musical timing, future task information, and piano state. As summarized in Figure 1, the complete observation has 2106 dimensions and consists of the following components:

(4) $$s_{t}=\left[q_{t},{\dot{q}}_{t},a_{t-1},p_{t},g_{t},f_{t},k_{t}\right],$$

where $q_{t}$ represents joint positions, ${\dot{q}}_{t}$ represents joint velocities, $a_{t-1}$ is the previous action, $p_{t}$ is the musical phase encoding, $g_{t}$ is the future key-target window, $f_{t}$ is the future fingering window, and $k_{t}$ is the current piano-key state.

Specifically, the observation includes 21 joint positions, 21 joint velocities, 21 previous action values, 2 phase features, 1848 future key-target features, 105 future fingering features, and 88 current piano-key states. The future key-target component corresponds to a 21-step lookahead window over 88 keys: 21×88=1848.

The future fingering component corresponds to a 21-step lookahead window over five fingers.

This lookahead design is important for piano playing because the robot must often prepare its hand before the next note occurs [12,13]. Human pianists also rely on anticipatory hand shaping and finger preparation, especially when performing chords, octaves, and rapid note sequences [1,2]. Fine-scale finger preparation is also related to the neural coordination of intrinsic and extrinsic hand muscles during single-finger pressing [5]. Therefore, this observation design is consistent with the biomimetic motivation of the study.

### 2.6. Action Space and Low-Level Position Control

The action vector is defined as $a_{t}\in {\left[-1,1\right]}^{21}$.

The 21 action dimensions correspond to wrist, finger, and forearm motion components:

(5) $$a_{t}=\left[a_{wrist},a_{finger},a_{forearm}\right],$$

where $a_{wrist}$ has 2 dimensions, $a_{finger}$ has 16 dimensions, and $a_{forearm}$ has 3 dimensions.

In simulation, the normalized action is first clipped to the interval $\left[-1,1\right]$. It is then linearly mapped to the physical joint-position limits:

(6) $$q_{target}=q_{min}+\frac{a_{t}+1}{2}\left(q_{max}-q_{min}\right).$$

The mapped joint targets are interpolated during the 10 physics substeps within each control interval and then applied to MuJoCo position actuators.

In real-world deployment, the same action-to-position mapping is used. The desired joint positions are sent to the hardware interface, and the low-level controller tracks them using PD position control. For each joint, the tracking command can be expressed as

(7) $$u=K_{p}\left(q_{target}-q\right)+K_{d}\left({\dot{q}}_{target}-\dot{q}\right),$$

where $q_{target}$ is the desired joint angle, q is the measured joint angle from the joint-angle sensor, and $K_{p}$ and $K_{d}$ are proportional and derivative gains. In the actual implementation, the PD controller is embedded in the joint servo layer, which converts position errors into motor commands. This low-level feedback loop is essential for compensating small mechanical errors and improving the stability of real key presses.

### 2.7. Soft Actor–Critic Policy Learning

The policy is trained using Soft Actor–Critic (SAC). SAC is suitable for this problem because it supports continuous action spaces and encourages exploration through entropy regularization.

The actor network maps the 2106-dimensional observation vector to a Tanh-Gaussian action distribution. It consists of two hidden layers with 256 units and GELU activation. The actor outputs the mean vector μ(s) and log standard deviation vector logσ(s), both with 21 dimensions. During training, the action is sampled from the Gaussian distribution and squashed through a hyperbolic tangent function:

(8) a=tanh(μ(s)+σ(s)ϵ),

where ϵ is sampled from a standard normal distribution. During deployment, deterministic inference is used:

(9) $$a=tanh\left(\mu \left(s\right)\right),$$

The critic contains two Q networks, $Q_{1}(s,a)$ and $Q_{2}(s,a)$. Each critic receives the concatenated state-action vector with dimension 2127 and outputs a scalar Q value. The twin-critic structure reduces overestimation bias by using the minimum of the two Q values in the target.

The critic target is computed as

(10) $$y=r+\gamma (1-d)\left[min(Q_{1}^{\prime }(s^{\prime },a^{\prime }),Q_{2}^{\prime }(s^{\prime },a^{\prime }))-\alpha log\pi (a^{\prime }|s^{\prime })\right],$$

where d indicates episode termination, γ is the discount factor, and α is the entropy temperature. The critic loss is the mean-squared error between the predicted Q values and the target. The actor is optimized to maximize

(11) $$E\left[Q(s,\tilde{a})-\alpha log\pi (\tilde{a}|s)\right].$$

The entropy coefficient is automatically tuned using a target entropy of H=−0.5|A|=−10.5, where |A|=21 is the action dimension. Target networks are updated using Polyak averaging.

For reproducibility, the reported implementation uses two online critics and two target critics, the minimum double-Q target, mean-squared critic loss, automatic entropy tuning with target entropy −0.5 × action dimension (−10.5 for the 21-dimensional action space), Polyak target updates, and critic networks with Dropout (0.01), LayerNorm, and GELU activations. Table 2 reports the principal optimization parameters used in the experiments. Gradient clipping is supported by the implementation but was disabled in the reported runs (grad_norm_clip = 0.0).

### 2.8. Two-Stage Reinforcement Learning Curriculum

A central part of the proposed method is the two-stage curriculum shown in Figure 1. The motivation is that accurate piano playing and physically smooth motion are both necessary, but optimizing them simultaneously from the beginning can make exploration difficult. If the regularization terms are too strong at the early stage, the agent may fail to discover effective key-pressing behaviors. Conversely, if regularization is always weak, the final policy may produce abrupt or unsafe actions that are difficult to deploy on hardware.

Therefore, the training procedure is divided into two stages.

**Stage 1: Key-Pressing Acquisition**

Stage 1 focuses on learning which keys to press and when to press them. Regularization terms related to power, velocity, and acceleration are weakened. This allows the agent to explore more freely and rapidly increase frame-wise key-state Recall and F1 score. The main objective of this stage is to establish a reliable mapping from future musical targets and fingering information to coordinated finger motions.

**Stage 2: Motion Refinement**

Stage 2 resumes from the corresponding Stage 1 network checkpoint. The network weights are restored, whereas the replay memory and optimizer states are not carried over. In this stage, the key-pressing and fingering rewards are strengthened, while physical regularization terms are restored or increased. Additional constraints, such as finger-speed limits, are activated. The goal is to preserve high key-state accuracy while regularizing command variation, discouraging undesirable contacts, and improving readiness for real-world deployment [14,18,19,20,27].

This curriculum resembles human motor learning in a broad sense: a performer first learns to hit the correct notes and later refines timing, smoothness, efficiency, and expressiveness.

### 2.9. Reward Function

The total reward is formulated as a weighted sum of task-oriented rewards and regularization terms:

(12) $$r_{\mathrm{total}}(t)=\Delta t\sum_{i}w_{i}r_{i}(t),$$

where $w_{i}$ is the weight of the i-th reward component and Δt=0.025 s is the control timestep.

The task reward comprises two main components: key pressing and fingering. The key-pressing reward is defined as

(13) $$r_{\mathrm{key}}=0.6r_{\mathrm{on}}+0.4r_{\mathrm{off}},$$

Here, $r_{\mathrm{on}}$ measures whether the target keys are pressed to the required depth using a tolerance-based function, while $r_{\mathrm{off}}$ penalises unintended key presses via an exponential penalty over the number of wrong keys:

(14) $$r_{\mathrm{off}}=exp(-1.25N_{\mathrm{wrong}}),$$

where $N_{\mathrm{wrong}}$ is the number of non-target keys activated at the current control step. Since chords require simultaneous pressing of multiple keys, we assign additional weights to chord events: two-key events receive a multiplier of 1.08, and events with three or more keys receive 1.15. This weighting scheme improves learning stability for multi-key actions.

The fingering reward encourages the assigned fingers to move towards their corresponding target keys. For each active finger, the distance from the fingertip to the key is evaluated using a Gaussian tolerance function, with a success bound of 0–0.01 m and a margin of 0.1 m. The reward is then averaged over all active fingers at the current step. This design explicitly enforces finger-to-key correspondence and encourages anatomically structured fingering behaviour rather than key activation through arbitrary finger assignments.

In addition to these task-oriented rewards, we introduce several regularisation terms designed to penalise excessive positive mechanical power, rapid command changes, and undesirable contacts, thereby regularising the policy outputs for sim-to-real deployment.

Power regularisation penalises excessive positive mechanical power of the forearm, wrist, and fingers. It is defined as

(15) $$r_{\mathrm{power}}=k\sum_{j}max(\tau_{j}{\dot{q}}_{j},0),$$

where $\tau_{j}$ and ${\dot{q}}_{j}$ are the joint torque and joint velocity of the j-th joint, respectively, and k<0 determines the penalty strength. The coefficients are set to $-1\times 10^{-3}$, $-1\times 10^{-2}$, and $-5\times 10^{-3}$ for the forearm, wrist, and finger power penalties, respectively.

Action velocity penalties suppress rapid command changes. For a group of action dimensions, the penalty is computed as

(16) $$r_{\mathrm{vel}}=k\sum_{j}\left|\frac{a_{t,j}-a_{t-1,j}}{\Delta t}\right|,$$

where $a_{t,j}$ is the j-th action component at timestep t. The coefficients for forearm, wrist, and finger velocity penalties are $-2\times 10^{-2}$, $-5\times 10^{-3}$, and $-2\times 10^{-4}$, respectively.

Action acceleration penalties further reduce abrupt changes in action velocity:

(17) $$r_{\mathrm{acc}}=k\sum_{j}\left|\frac{a_{t,j}-2a_{t-1,j}+a_{t-2,j}}{\Delta t^{2}}\right|,$$

with coefficients $-2\times 10^{-3}$, $-3\times 10^{-4}$, and $-1\times 10^{-5}$ for the forearm, wrist, and finger, respectively.

In Stage 2, an additional finger-speed limit is activated to constrain excessively fast finger commands:

(18) $$r_{\mathrm{fs}}=k\sum_{j}max,\;\left(|\frac{a_{t,j}-a_{t-1,j}}{\Delta t}|-v_{max},0\right),$$

where the summation applies only to finger joints, k=−0.05, and $v_{max}=6.5\;\mathrm{rad}/s$. This one-sided penalty does not affect normal finger motion but only penalises the portion exceeding the speed limit.

The collision penalty discourages unsafe self-collisions and undesired contacts:

(19) $$r_{\mathrm{col}}=0.2(1-N_{\mathrm{contact}}),$$

where $N_{\mathrm{contact}}$ is the number of undesired contact pairs.

The natural-stretch reward penalises deviations from a default relaxed hand posture:

(20) $$r_{\mathrm{nat}}=-4\times 10^{-3}\sum_{j}|a_{j}-a_{\mathrm{base},j}|,$$

where $a_{\mathrm{base}}$ is the predefined natural configuration and the summation runs over non-forearm action dimensions.

A positive wrist-pitch reward encourages a favourable wrist posture during pressing: $r_{\mathrm{wp}}=0.05$ if $a_{wrist\_pitch}>0$, otherwise 0.

Finally, a forward-press constraint penalises hyperextended pressing postures: for each finger, a penalty of −0.1 is applied when the equivalent pressing angle exceeds π/2.

Collectively, these regularisation terms are designed to discourage excessive power-related objective values, abrupt command changes, undesirable contacts, and implausible postures. During Stage 1, the regularisation weights are reduced to facilitate exploration and key-press acquisition. In Stage 2, the weights are increased and the finger-speed limit is activated. Table 3 reports the stage-dependent external reward weights used in the experiments.

### 2.10. Evaluation Metrics

The main evaluation metrics are frame-wise key-state Precision, Recall, and F1 score. The target MIDI sequence and the robot-played MIDI sequence are discretized at the 25 ms control interval. At each control step, the 88 piano keys are represented as binary activation states. A true positive is counted when a key is active in both the target grid and the played grid at the same time step. A false positive is counted when a key is active in the played grid but not in the target grid, whereas a false negative is counted when a target key is active but not successfully played at the corresponding time step. Because sustained notes occupy multiple consecutive frames, they contribute multiple key-state samples to these metrics.

Precision is defined as

(21) $$Precision=\frac{TP}{TP+FP}.$$

Recall is defined as

(22) $$Recall=\frac{TP}{TP+FN}.$$

The F1 score is defined as

(23) $$F1=2\cdot \frac{Precision\cdot Recall}{Precision+Recall}.$$

Precision measures the fraction of active played key-state frames that match the target, Recall measures the fraction of target active key-state frames reproduced by the robot, and F1 provides a balanced measure of both. These metrics are used in simulation and real-world experiments. They should not be interpreted as event-based note-onset scores, because small timing offsets can affect several consecutive 25 ms samples.

### 2.11. Sim-to-Real Deployment Pipeline

The deployment process is summarized in the lower part of Figure 1. After training, the system saves checkpoints containing the policy, twin critics, entropy coefficient, and environment parameters. The policy is then evaluated in simulation using frame-wise key-state Precision, Recall, and F1 score.

The trained policy can be exported through a recorder. The recorder stores time stamps, 21-dimensional actions, policy actions, joint positions, joint velocities, and piano activation states. Two export modes are supported. In the first mode, the policy performs step-by-step online inference within the simulation/recording environment and records the resulting actions. In the second mode, the recorded action sequence is saved and later replayed open-loop on the physical system. The term online inference here refers to the simulation/recording stage and should not be confused with high-level closed-loop inference on the physical robot.

For the reported real-world deployment, the high-level policy-generated sequence is replayed open-loop. The exported action sequence is executed at 40 Hz; before execution, the actions are clipped, mapped to joint-position limits, checked against command velocity and acceleration limits, aligned with the MIDI phase clock, and interpolated to 100 Hz for hardware playback. This high-level open-loop execution is distinct from the low-level joint servos, which continuously use joint-angle feedback for position tracking.

The hardware interface layer includes functions for connection, homing, action application, timing control, and sensor reading. The final joint tracking is performed by the low-level position servo with joint-angle feedback and PD control [14,20,21,22].

## 3. Results

### 3.1. Baseline Performance of the Original RoboPianist Training Pipeline

We first evaluated the original RoboPianist training pipeline on the same three 30 s right-hand excerpts used for the proposed method. Unless otherwise stated, each simulation experiment for both the baseline and the proposed method was repeated three times with different random seeds. The solid learning curves represent the mean across the three independent runs, and the shaded regions denote ±1 standard deviation (SD) around the mean at each evaluation point.

**Figure 3:**
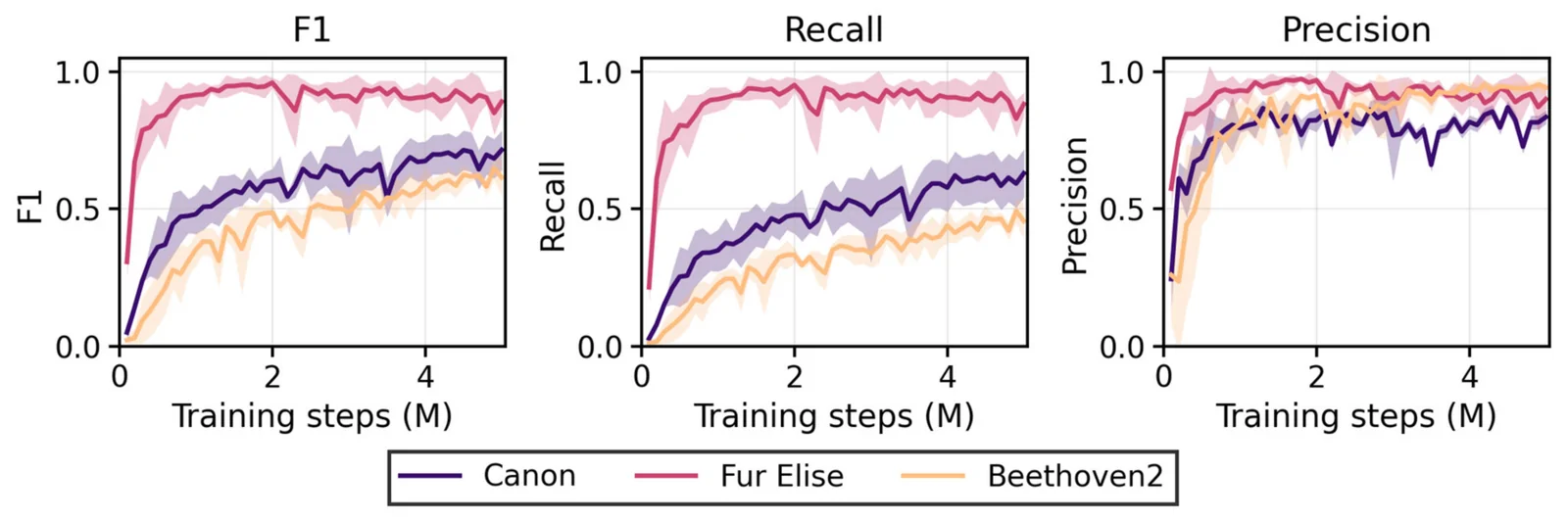
Baseline training performance. F1, Recall, and Precision curves of the original RoboPianist pipeline on three piano excerpts. Solid curves represent the mean across three independent runs; shaded regions denote ±1 standard deviation (SD) around the mean.

The baseline results are shown in Figure 3. For the relatively simple Für Elise excerpt, the baseline reaches an F1 score of approximately 0.98 after approximately 5 million single-environment interaction steps. For the more difficult Canon and Beethoven excerpts, the baseline reaches F1 scores of approximately 0.70 and 0.65, respectively. As defined in Section 2.4, this step count is not directly comparable with the vectorized-step count used by the proposed implementation. The two implementations also differ in robotic/control stack, control frequency, environment parallelization, and optimizer-update schedule; the comparison below is therefore interpreted at the task-performance level rather than as a normalized sample-efficiency or computational-efficiency benchmark.

These results indicate that the original training pipeline can handle relatively simple melodic passages but struggles with more complex musical structures. The performance degradation is especially clear in passages requiring repeated notes, denser temporal patterns, or more demanding finger coordination. This observation motivates the structured task representation and two-stage curriculum investigated in the proposed framework.

### 3.2. Simulation Performance of the Proposed Two-Stage Curriculum

**Figure 4:**
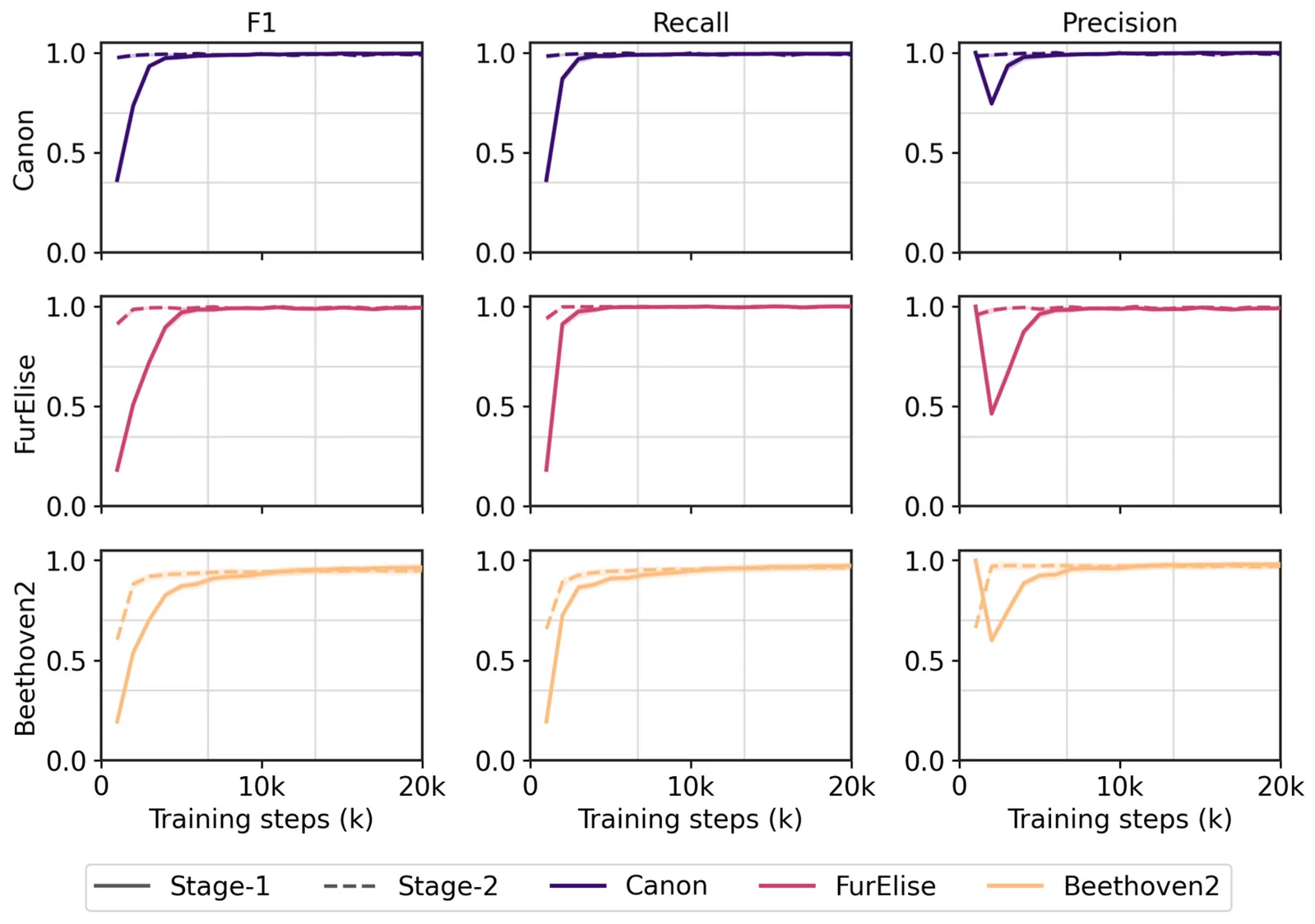
Training performance of the proposed method. Simulation learning curves of the two-stage curriculum on *Für Elise*, *Canon*, and Beethoven excerpts. Solid curves represent the mean across three independent runs; shaded regions denote ±1 standard deviation (SD) around the mean.

The learning curves of the proposed method are shown in Figure 4. For Für Elise, Stage 1 reaches an F1 score above 0.99 within approximately 5000 vectorized environment steps, corresponding nominally to approximately 1.28 million individual transitions across the 256 parallel environments. Stage 2 maintains an F1 score above 0.99 after increasing the regularization weights. A similar trend is observed for Canon, where the F1 score converges above 0.99 within several thousand vectorized steps.

The Beethoven excerpt is considerably more difficult. It contains faster transitions and more demanding finger coordination. Nevertheless, the proposed method achieves an F1 score of approximately 0.96 in Stage 1 after 20,000 vectorized environment steps, corresponding nominally to approximately 5.12 million individual transitions. After Stage 2 refinement, the policy converges to an F1 score of approximately 0.945. Although the final F1 score is lower than those of Für Elise and Canon, it remains higher than the baseline task-level result shown in Figure 3; this comparison concerns achieved task performance rather than raw step-count efficiency.

The Recall and Precision curves in Figure 4 provide additional insight. Stage 1 rapidly improves Recall, indicating that the robot quickly learns to cover the target notes. Stage 2 maintains high Precision while refining the motion. This suggests that the curriculum successfully separates note acquisition from physical motion refinement.

### 3.3. Evolution of Weighted Regularization Terms During Stage 2

**Figure 5:**
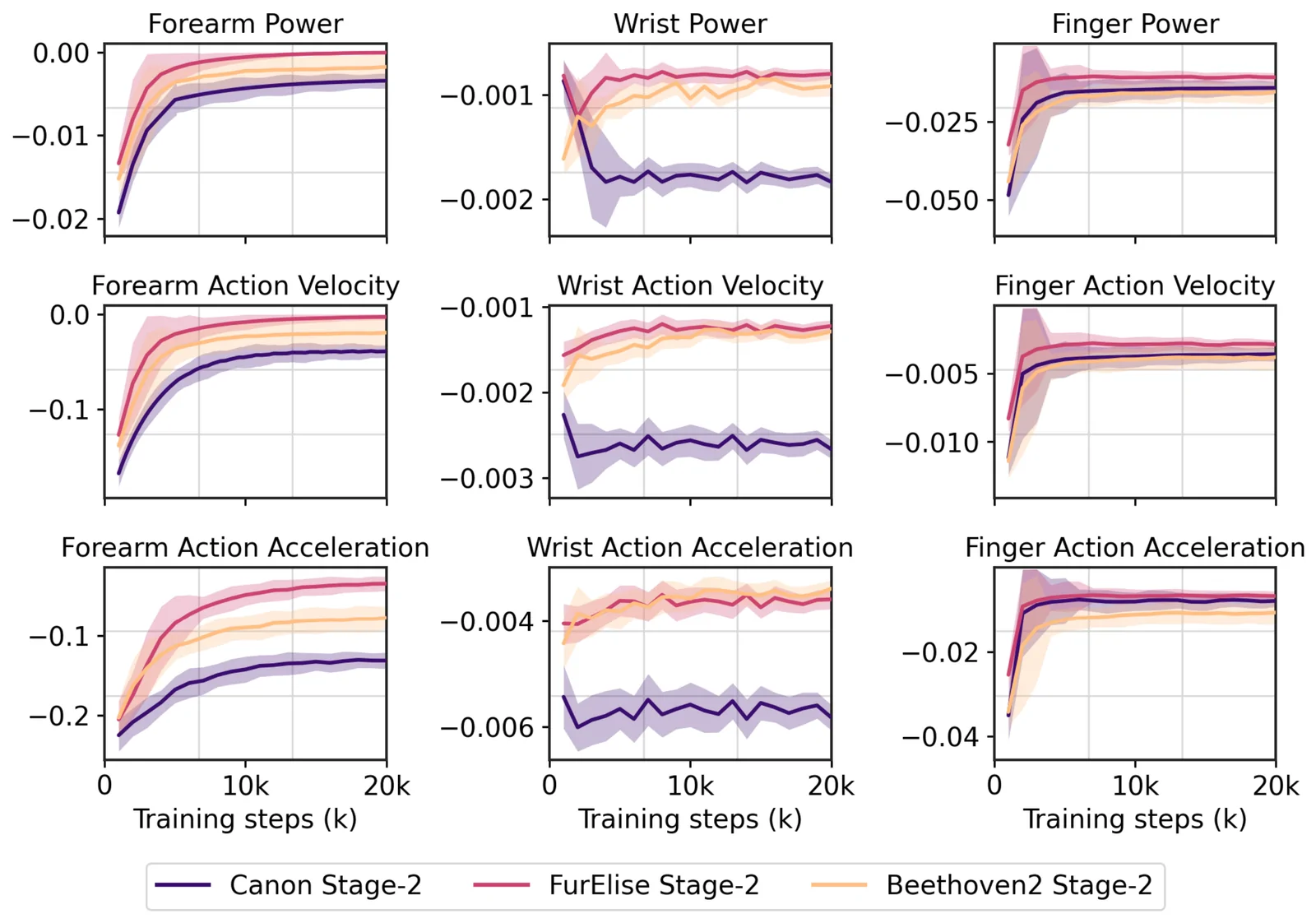
Weighted regularization-term contributions during Stage 2 training. The plotted values are reward/penalty objective contributions averaged over evaluation frames and should not be interpreted as direct physical measurements of energy consumption, safety, or trajectory smoothness.

High key-state accuracy alone is insufficient for physical robotic piano playing. To examine how the Stage 2 regularization objectives evolve during optimization, we evaluated the weighted reward/penalty contributions associated with the regularization terms, as shown in Figure 5. These plotted quantities are objective-function contributions rather than direct physical measurements of energy consumption, safety, or motion smoothness.

The monitored quantities include weighted contributions associated with forearm, wrist, and finger power, action velocity, and action acceleration. Each plotted value includes the corresponding reward-function coefficient and RewardManager weight and is averaged over the evaluation frames. Their stabilization during Stage 2 indicates that these optimization terms become more stationary as training progresses; it does not by itself establish a reduction in physical energy consumption or a quantitative improvement in physical safety.

These regularization terms are intended to discourage abrupt command changes and large power-related objective values, which are undesirable for hardware deployment. Direct claims about physical energy efficiency, safety, or trajectory smoothness would require dedicated physical indicators such as measured mechanical power, RMS velocity/acceleration, or constraint-violation statistics; such measurements are outside the scope of the present revision.

### 3.4. Learning of Representative Piano Techniques

**Figure 6:**
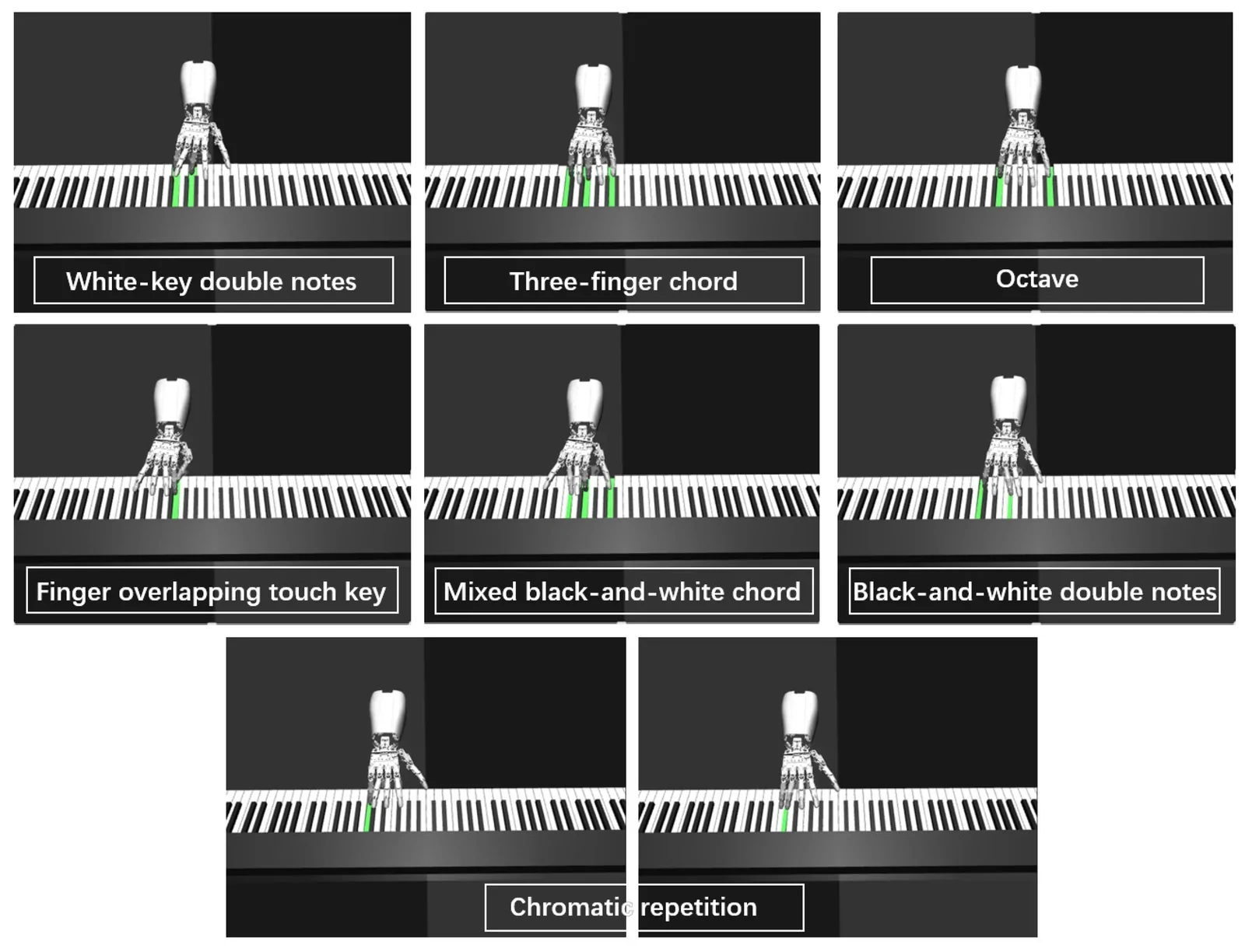
Piano techniques learned in simulation. Representative learned skills, including double notes, chords, octaves, overlapping touches, and chromatic repetitions.

One of the key motivations of this study is to evaluate whether a biomimetic robotic hand can learn representative piano-playing techniques requiring coordinated multi-finger control. Figure 6 shows several such techniques learned in simulation, including white-key double notes, three-finger chords, octaves, finger-overlapping key touches, mixed black-and-white-key chords, black-and-white double notes, and chromatic repetitions.

These skills are meaningful because they require different types of hand coordination. Double notes and chords require simultaneous multi-finger pressing. Octaves require larger spatial separation between fingers and stable wrist positioning. Mixed black-and-white-key chords require accurate vertical and horizontal fingertip placement due to the different heights and positions of white and black keys. Chromatic repetition requires rapid sequential finger movement with precise timing.

The successful execution of these techniques shows that the policy can generate coordinated multi-finger strategies spanning several fundamental piano-playing patterns [1,2,3,4,5,8]. Because the study does not include a quantitative comparison with human kinematics, posture trajectories, or timing strategies, these behaviors are described as biomimetically inspired rather than experimentally demonstrated human-like motion.

### 3.5. Ablation Study 1: Removing Joint Velocity Observation

**Figure 7:**
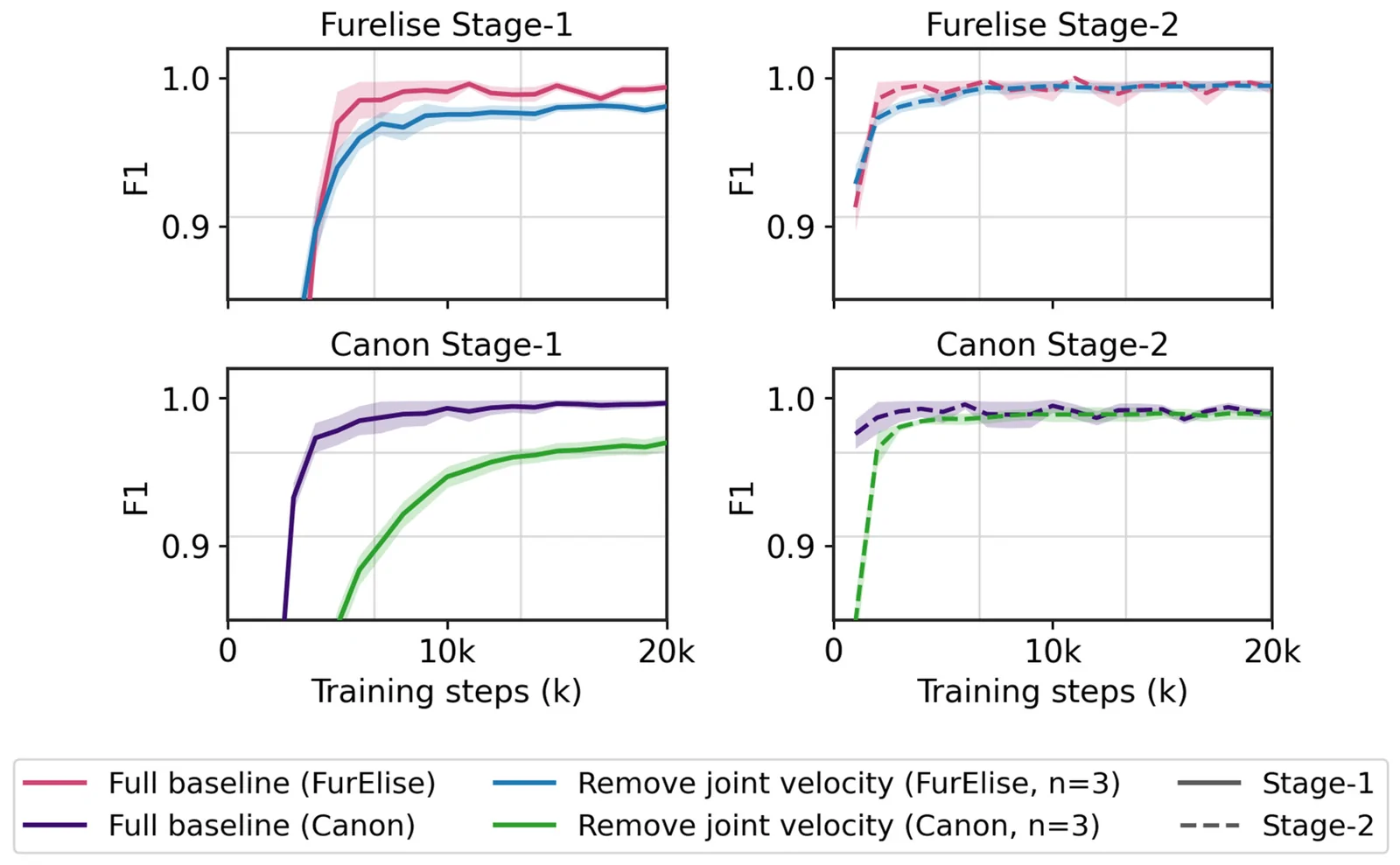
Ablation of joint velocity observation. Performance comparison between the full model and the variant without joint velocity observation. Solid curves represent the mean across three independent runs; shaded regions denote ±1 standard deviation (SD) around the mean.

The first ablation study evaluates the contribution of joint velocity information. The results are shown in Figure 7. For Für Elise, removing joint velocity information reduces the Stage 1 F1 score from approximately 0.995 to about 0.975, indicating that velocity feedback still contributes to the initial acquisition of accurate key-pressing behavior. However, after Stage 2 refinement, the performance only decreases slightly compared with the full model, suggesting that the policy can largely compensate for the missing velocity information during the motion-refinement stage.

A similar trend is observed for Canon. In Stage 1, removing joint velocity information reduces the F1 score from approximately 0.992 to about 0.96. This indicates that joint velocity is more helpful during the initial learning stage, especially for passages with denser note transitions. In Stage 2, however, the F1 score decreases only slightly and remains close to that of the full model. These results suggest that joint velocity information mainly improves Stage 1 learning efficiency and early key-press acquisition, while its effect on the final refined policy after Stage 2 is relatively limited.

### 3.6. Ablation Study 2: Removing Previous Action Observation

**Figure 8:**
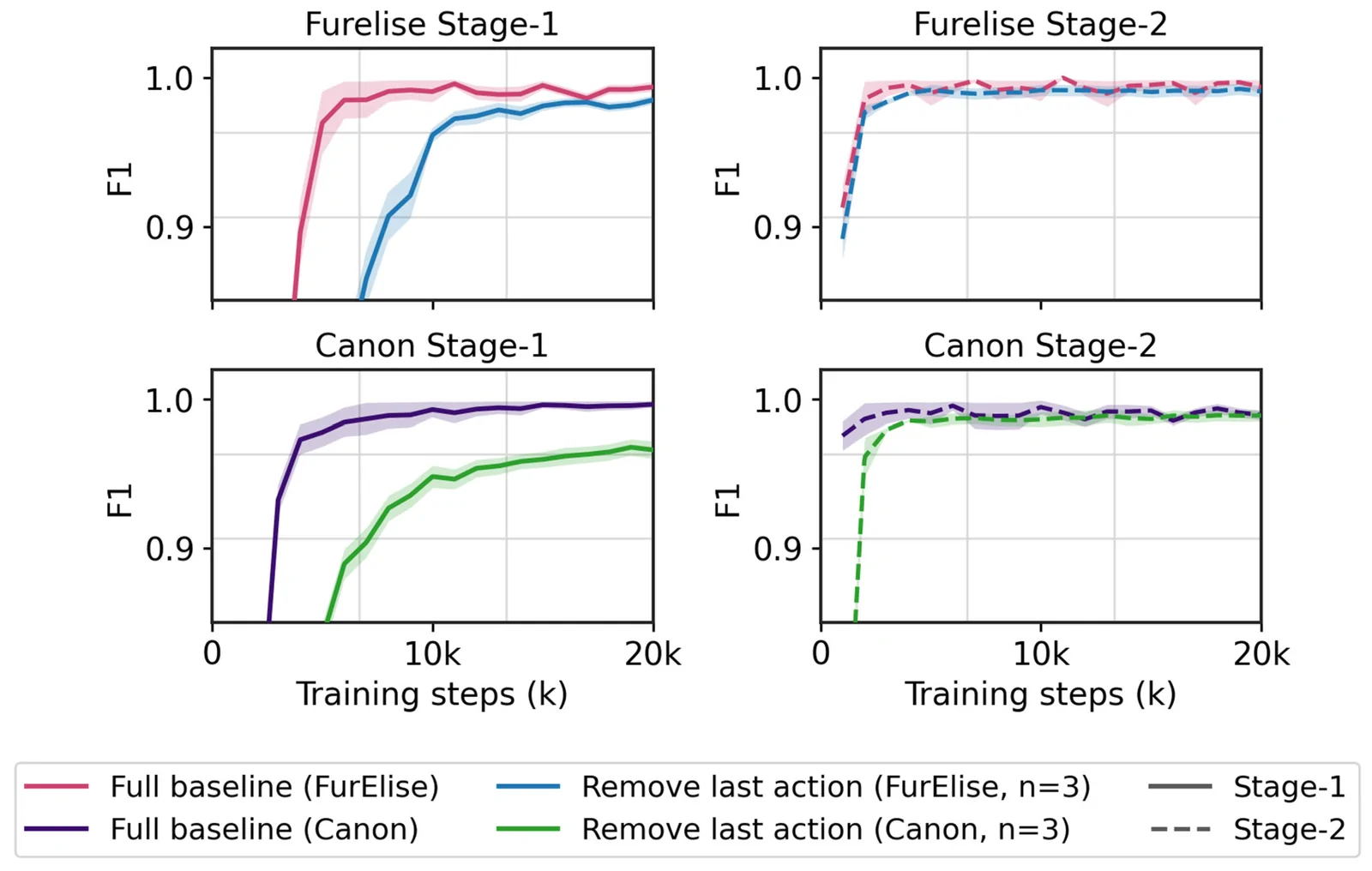
Ablation of previous action observation. Performance comparison between the full model and the variant without previous action input. Solid curves represent the mean across three independent runs; shaded regions denote ±1 standard deviation (SD) around the mean.

The second ablation study removes the previous action from the observation vector. The results are shown in Figure 8. For Für Elise, the Stage 1 F1 score decreases from approximately 0.995 to about 0.98, while the Stage 2 performance only shows a slight reduction compared with the full model. For Canon, the effect is more pronounced in Stage 1, where the F1 score decreases from approximately 0.992 to about 0.96. However, after Stage 2 refinement, the final F1 score also decreases only slightly.

The previous action provides short-term memory of the policy’s own motor command. This information helps the agent maintain action continuity, which is particularly useful for high-frequency multi-finger control and dense note transitions. Removing this component makes it more difficult for the policy to infer recent motion trends during the initial learning stage, resulting in lower Stage 1 performance. Nevertheless, the limited performance drop after Stage 2 suggests that the refinement stage can partially compensate for the missing previous-action information by improving motion regularity and stabilizing the learned policy.

### 3.7. Ablation Study 3: Removing the Two-Stage Curriculum

**Figure 9:**
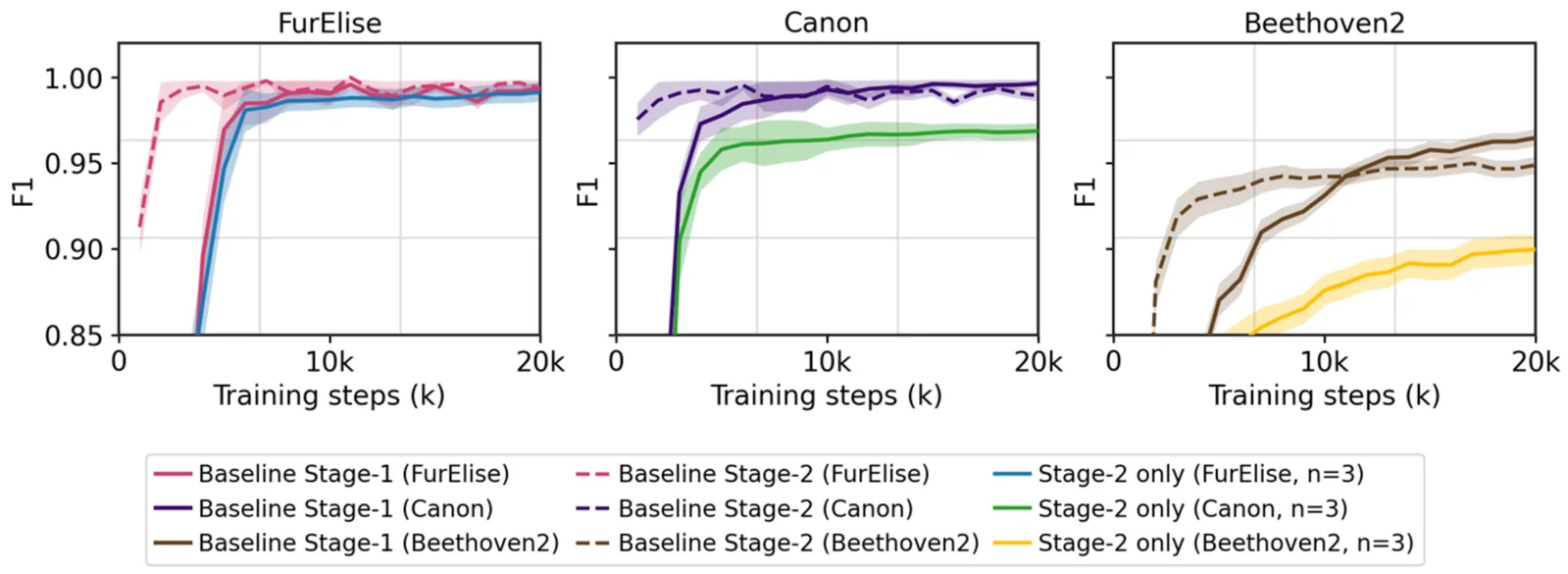
Ablation of two-stage curriculum learning. Performance comparison between curriculum learning and direct Stage-2 training. Solid curves represent the mean across three independent runs; shaded regions denote ±1 standard deviation (SD) around the mean.

The third ablation study evaluates the importance of the two-stage curriculum. In this experiment, Stage 2 is trained directly without first learning under the Stage 1 configuration. The results are shown in Figure 9.

For *Für Elise*, direct Stage 2 training has little influence, suggesting that simple passages can still be learned under stronger regularization. For *Canon*, the final F1 score decreases by approximately 0.03. For the Beethoven excerpt, the final F1 score decreases by approximately 0.05.

These results confirm that the two-stage curriculum is particularly beneficial for complex musical passages. Strong regularization from the beginning can restrict exploration and prevent the agent from discovering effective key-pressing strategies. By first learning the task under weaker regularization and then refining the motion under stronger constraints, the proposed curriculum improves both convergence and final performance.

### 3.8. Real-World Robotic Piano Performance

**Figure 10:**
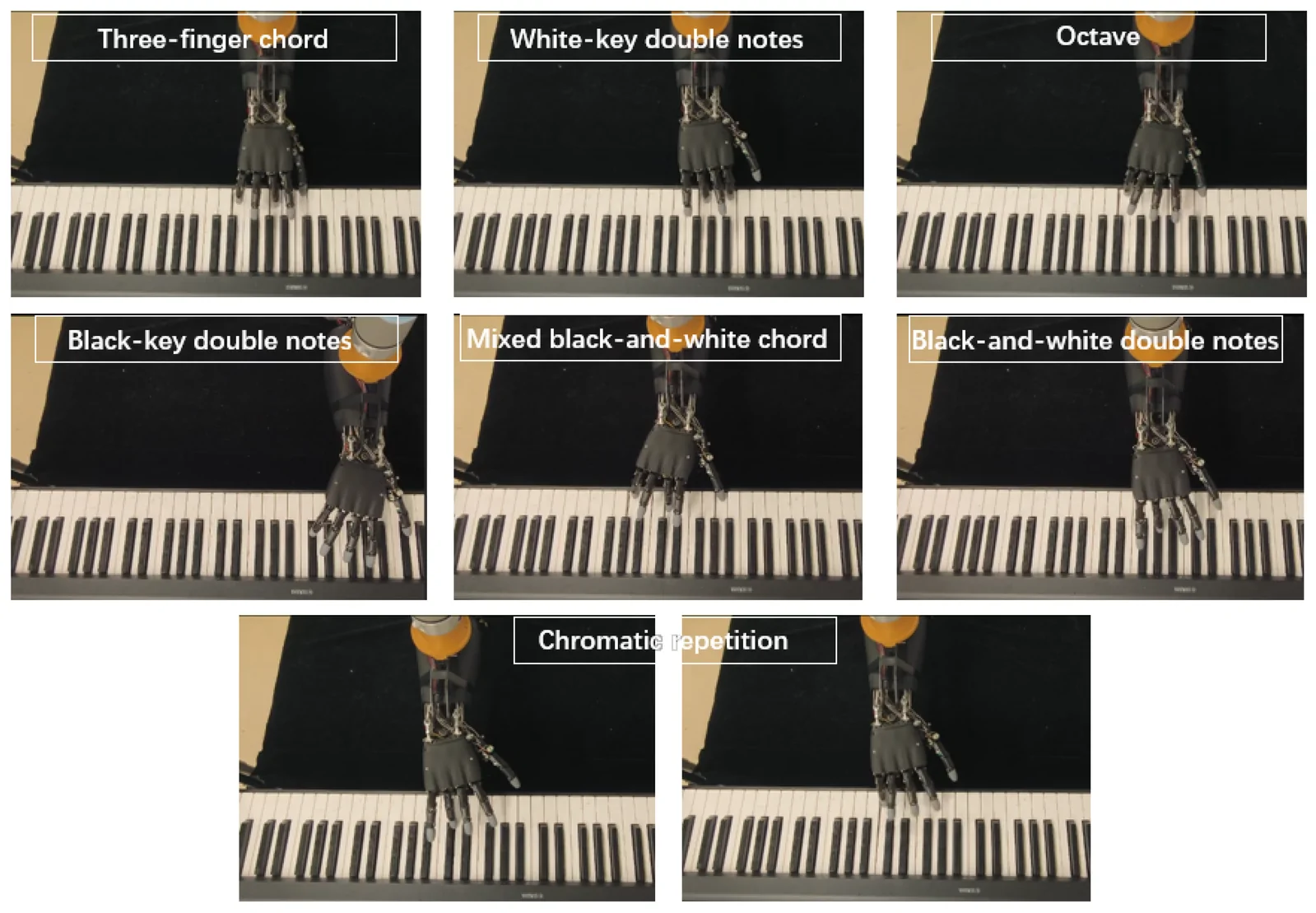
Real-world demonstrations of piano techniques. Physical execution of representative multi-finger piano skills on the robotic hand.

**Figure 11:**
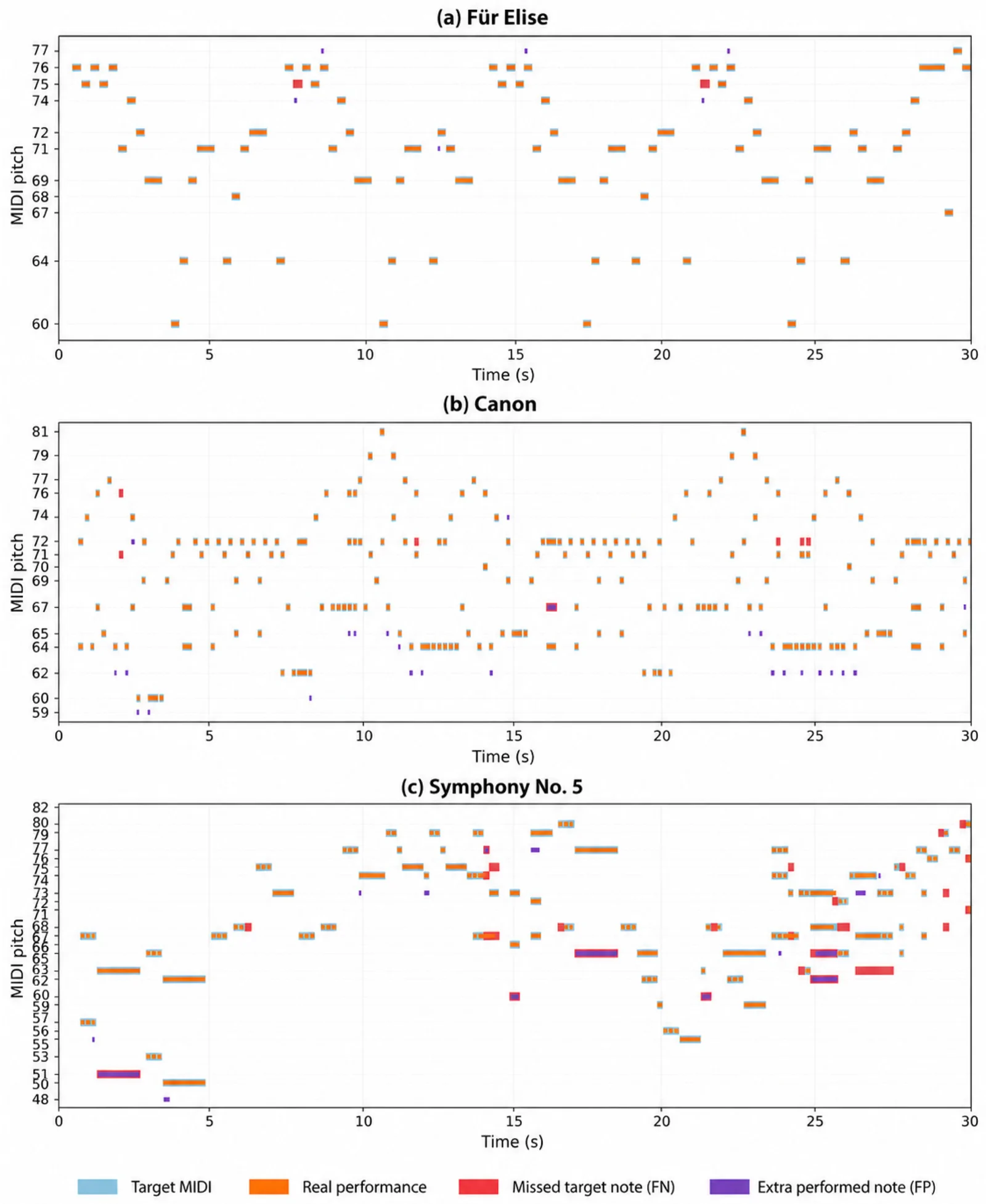
Pitch-time visualization of real-world performance. Rectangular note representations of the target MIDI sequence and the robot-played notes on the physical platform.

**Figure 12:**
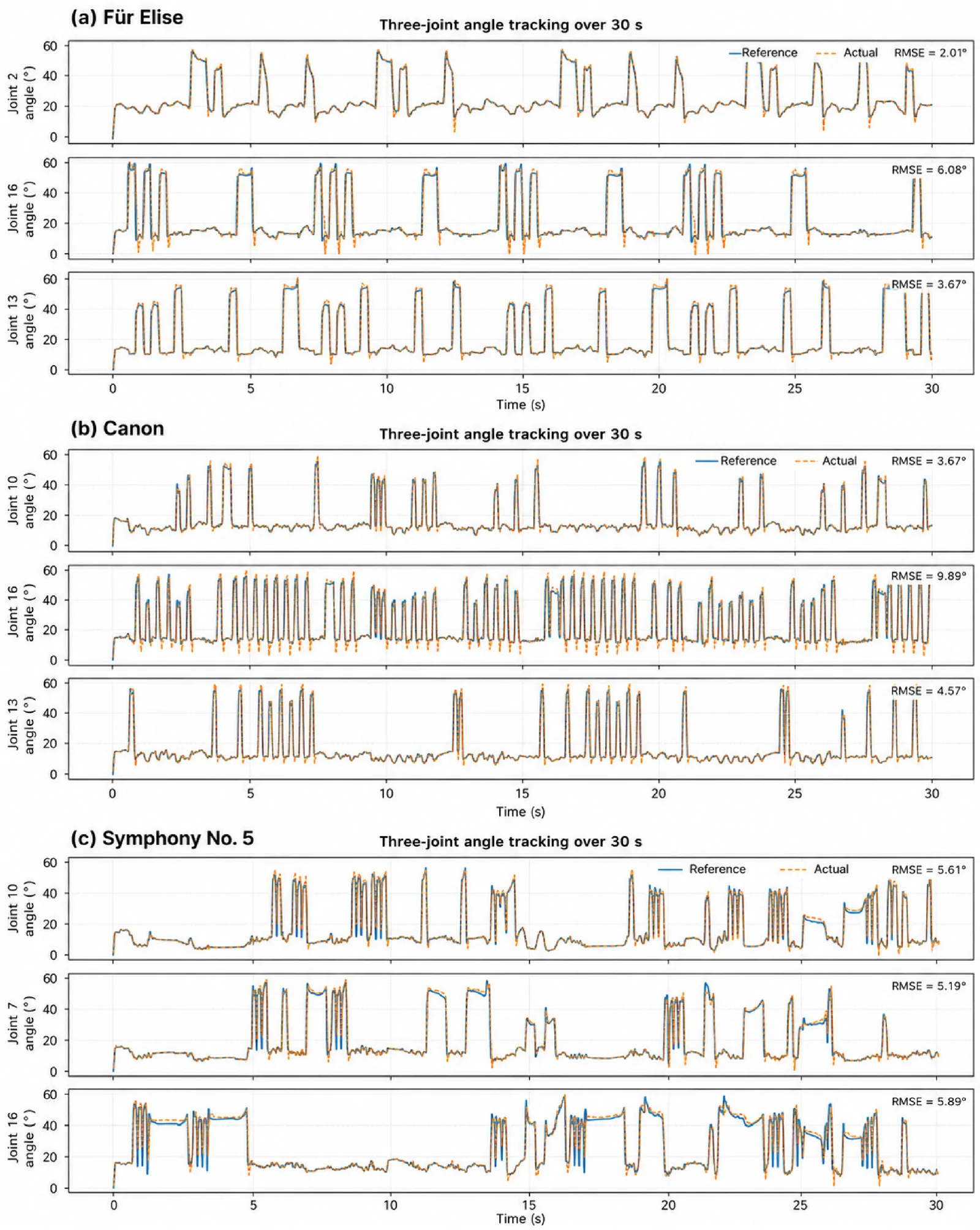
Joint-angle tracking performance during real-world piano playing. For each piece, the three joints with the largest target-motion amplitudes are selected for visualization. Solid curves denote target joint trajectories, dashed curves denote measured joint angles, and RMSE values are reported in each subplot.

After simulation training and action export, the policy-generated action trajectories were deployed on the physical robotic piano-playing system. One complete physical execution was conducted for each 30 s musical excerpt in the reported evaluation. The F1 scores, Figure 10, Figure 11 and Figure 12, and the corresponding segments of the Supplementary Video S1 refer to these single reported executions. The real-world performance is shown in Figure 10, Figure 11 and Figure 12.

Figure 10 presents physical demonstrations of representative piano techniques performed by the robotic hand. These techniques correspond to the musical skills discussed in the simulation analysis, including mixed black-and-white-key chords, overlapping finger contacts, rapid sequential patterns, three-finger chords, octaves, repeated notes, and dense multi-finger transitions. The results show that the learned motion patterns can be transferred from simulation to hardware and can produce recognizable coordinated multi-finger piano-playing patterns [20,21,22].

In the single reported physical execution of each excerpt, the robot achieves a frame-wise key-state F1 score of 0.95 on Für Elise, 0.91 on Canon, and 0.83 on the Beethoven excerpt. These values are lower than the best simulation results. The observed discrepancies are consistent with several plausible sim-to-real factors, including mechanical backlash, calibration error, friction differences, key-contact uncertainty, sensor noise, and the sensitivity of open-loop high-level playback; the present experiment does not isolate the contribution of each factor.

Figure 11 visualizes the pitch-time correspondence between the robot-played notes and the target MIDI sequence. In this figure, musical notes are represented as rectangular blocks in the pitch-time plane, allowing the target notes and the notes produced by the physical robot to be compared directly. The results indicate that the robot can reproduce most of the target note sequence with high pitch and timing consistency. The remaining errors mainly appear in dense or fast passages, where small timing deviations, incomplete key presses, or unintended neighboring-key contacts may lead to false positives or false negatives [12,21,22].

Figure 12 further shows the joint-angle tracking performance during real-world execution. For each of the three pieces, the three joints with the largest motion amplitudes in the target trajectory are automatically selected for visualization. The solid curves represent the target joint trajectories, whereas the dashed curves represent the measured joint angles from the physical robotic hand. The RMSE value is also reported in each subplot to quantify the tracking error.

The joint-angle tracking results show that the physical robotic hand generally follows the target trajectories during the reported 30 s executions. The measured trajectories are consistent with the target trajectories in terms of the main peaks, valleys, and overall temporal trends. Small tracking errors occur during rapid rising or falling motions and near sharp peaks, where slight delay, overshoot, or amplitude deviation can be observed. Such discrepancies are consistent with motor response delay, mechanical inertia, friction, transmission backlash, joint-angle sensor noise, and contact-force disturbances during fast key pressing. These results demonstrate low-level trajectory tracking within the reported executions but should not be interpreted as a statistical hardware-reliability assessment across repeated trials.

## 4. Discussion

The experimental results support the feasibility of the proposed two-stage reinforcement learning framework for biomimetic robotic piano playing. The framework is designed around three biologically inspired components—anticipatory observation, explicit fingering guidance, and progressive curriculum learning—which are used jointly to generate coordinated piano-playing behaviors without explicit motion imitation. The curriculum is examined directly through a dedicated ablation, whereas the independent causal contributions of the 21-step lookahead window and the fingering representation are not isolated in the present study. We therefore avoid attributing the overall performance gain to either component individually. The baseline and proposed implementations also define training steps differently and use different environment parallelization and update schedules; consequently, raw step counts are not interpreted as a direct sample-efficiency comparison. At the task-performance level, the proposed framework maintains high F1 scores on all three excerpts, including the denser Canon and Beethoven passages.

The two-stage curriculum is intended to balance key-state accuracy with progressively stronger regularization of the policy outputs. In Stage 1, weaker physical regularization allows broader exploration during key-press acquisition; in Stage 2, stronger regularization discourages large power-related objective values, abrupt action changes, undesirable contacts, and excessive finger speeds. From a motor-learning perspective, this coarse-to-refined progression is broadly analogous to learning task accuracy before refining movement quality [1,2]. Figure 5 should, however, be interpreted specifically as the evolution of weighted regularization objective terms. Their stabilization documents training behavior but does not by itself demonstrate lower physical energy consumption, greater safety, or quantitatively smoother hardware motion.

The ablation studies clarify the roles of joint-velocity observation, previous-action input, and the two-stage curriculum. Removing joint velocity or previous-action information mainly reduces Stage 1 performance, especially for Canon, whereas the gap becomes smaller after Stage 2 refinement. The curriculum ablation indicates that direct training under the stronger Stage 2 regularization is less effective for the more complex excerpts. These results support the staged optimization strategy, while the individual causal contributions of the 21-step lookahead representation, predefined fingering guidance, and individual SAC implementation choices are not isolated by the present experiments.

The physical experiments demonstrate that policy-generated trajectories can be executed on the Beyond Hand piano-playing platform. In the single reported execution of each excerpt, the robot achieves frame-wise key-state F1 scores of 0.95, 0.91, and 0.83 on Für Elise, Canon, and Beethoven, respectively. Figure 10 and Figure 11 show representative multi-finger techniques and pitch-time correspondence within these complete executions, while Figure 12 shows that the low-level position servos generally track the exported target trajectories. The current high-level deployment is explicitly open-loop: the learned action sequence is generated in advance and does not perform online note-error correction during physical playing. This should be distinguished from the low-level joint servos, which continuously use joint-angle feedback for PD position tracking. The physical results therefore establish feasibility of trajectory transfer and execution, not closed-loop high-level policy control or across-trial repeatability.

The present physical evaluation should therefore be interpreted as a feasibility demonstration rather than a statistical repeatability study. Because each musical excerpt was executed once in the reported physical evaluation, trial-to-trial robustness was not quantified. At the high level, open-loop trajectory playback can be sensitive to small joint-zero offsets, keyboard alignment errors, actuator temperature, transmission friction, fingertip–key friction, surface compliance, and contact-location changes. Key-surface friction is particularly relevant because it changes tangential slip and the effective fingertip contact location during pressing. The low-level joint servo feedback compensates for local joint-position tracking errors, but it cannot correct high-level note-selection or contact errors once the action sequence has been exported. Future work will therefore focus on online high-level inference with MIDI, tactile, vision, or audio feedback and disturbance-aware adaptation. Related observer-enhanced actor–critic control [33] and physical-performance policy-optimization self-learning control [34] provide complementary directions for disturbance estimation and online controller refinement under uncertain dynamics.

From the perspective of biomimetics, the present results show that a biomimetic hand embodiment, structured musical task representation, fingering constraints, and reinforcement learning can jointly generate coordinated piano-playing behaviors. The three design principles introduced in Section 1 are reflected in the implementation: future key targets support anticipatory preparation; the fingering grid provides an explicit finger-to-key prior; and the two-stage curriculum separates initial task acquisition from later motion regularization. These design choices are inspired by aspects of human piano biomechanics and motor learning, but the present study does not quantitatively compare robotic and human hand kinematics, posture trajectories, or timing strategies. We therefore describe the resulting behaviors as biomimetically or human-inspired rather than as experimentally demonstrated human-like motion. Robotic piano playing remains a useful benchmark because it combines temporal precision, multi-finger coordination, contact-rich interaction, and anticipatory motion planning.

The current framework is conditioned on predefined fingering assignments and does not autonomously select fingering. Consequently, a non-standard or sub-optimal fingering changes both the structured observation and the finger-to-key reward target, and performance may degrade if the assignment is inconsistent with the one used to train a policy. Sensitivity to alternative fingering choices was not systematically isolated in the present study. Likewise, the individual causal contribution of the 21-step lookahead window was not independently ablated. These components should therefore be interpreted as jointly used design choices rather than individually validated sources of performance improvement. Future work could learn fingering jointly with control, generate multiple feasible fingering candidates, or condition a single policy on alternative fingering plans.

Several limitations remain. First, the current experiments focus on 30 s right-hand excerpts rather than full-length two-hand piano performance. Extending the system to two hands would introduce additional challenges, including inter-hand coordination, larger workspace transitions, pedaling, and more complex fingering strategies. Second, the present physical evaluation uses open-loop high-level trajectory playback, and only one complete physical execution was reported for each excerpt. The reported F1 values, Figure 10, Figure 11 and Figure 12, and Supplementary Video S1 therefore characterize those executions and do not quantify trial-to-trial repeatability. Repeated hardware experiments under controlled variations in calibration, temperature, friction, and contact conditions will be required for a statistical robustness assessment. Third, the current framework relies on predefined fingering annotations; sensitivity to alternative or sub-optimal fingering assignments was not systematically evaluated. Fourth, the current frame-wise key-state evaluation counts sustained notes across multiple 25 ms samples and can be sensitive to small timing shifts. Event-based onset matching with an explicit temporal tolerance, key-duration analysis, key velocity, articulation, and musical expressiveness would provide complementary evaluations in future work. Finally, accurate calibration between the simulated piano, the physical keyboard, and the robotic hand remains important. Future closed-loop high-level control with MIDI, tactile, vision, or audio feedback, together with adaptive contact/friction modeling, is expected to improve robustness and enable online error correction.

## 5. Conclusions

This paper presented a biomimetic dexterous robotic piano-playing framework based on a two-stage reinforcement learning curriculum. The system converts MIDI data into target-key and fingering grids, trains a policy in a parallel MJLab simulation environment, and deploys the learned action trajectories to a physical robotic piano-playing platform consisting of a UR5 robotic arm, a Beyond Hand dexterous hand, and a Yamaha digital piano.

The proposed method integrates anticipatory musical observation, fingering-guided control, Soft Actor–Critic policy learning, multi-component reward design, a two-stage curriculum, and sim-to-real deployment using open-loop high-level trajectory playback with low-level PD position control. Stage 1 emphasizes key-pressing acquisition under weaker regularization, whereas Stage 2 increases power-, velocity-, acceleration-, collision-, posture-, and finger-speed-related regularization. The curriculum therefore separates initial task acquisition from subsequent regularization of the policy outputs.

Simulation experiments on 30 s right-hand excerpts from Für Elise, Canon, and Beethoven’s Symphony No. 5 show that, under their respective implementations, the proposed framework achieves higher task-level F1 scores than the original RoboPianist baseline on the three evaluated excerpts, particularly on Canon and Beethoven’s Symphony No. 5. Because the two implementations differ in robotic/control stack, control frequency, environment parallelization, and optimizer-update schedule, this task-level comparison should not be interpreted as a normalized sample-efficiency or computational-efficiency benchmark. Across three independent training runs, the proposed method achieves F1 scores above 0.99 on Für Elise and Canon and approximately 0.945 on the more difficult Beethoven excerpt. The ablation studies directly examine joint-velocity observation, previous-action input, and the two-stage curriculum; the individual effects of the lookahead and fingering representations, as well as separate SAC implementation choices, are not isolated in the present experiments.

In the reported single physical execution of each excerpt, the learned action trajectories were successfully transferred to the physical robotic platform, yielding frame-wise key-state F1 scores of 0.95, 0.91, and 0.83 on Für Elise, Canon, and Beethoven, respectively. Pitch-time MIDI visualization and joint-angle tracking demonstrate execution of the exported trajectories within these 30 s performances. Because the high-level trajectory is replayed open-loop and no repeated-trial statistics are reported, the physical results should be interpreted as a feasibility demonstration rather than evidence of high-level closed-loop error correction or statistical hardware repeatability.

The results demonstrate that a biomimetic robotic hand can learn representative complex piano-playing techniques, including double notes, chords, octaves, mixed black-and-white-key patterns, overlapping finger actions, repeated notes, and rapid sequential movements, without relying on human motion demonstration trajectories. These behaviors are biomimetically inspired; the present study does not establish quantitative equivalence to human hand kinematics. The work provides a promising approach for contact-rich biomimetic dexterous manipulation and supports robotic piano playing as a benchmark for multi-finger coordination and sim-to-real dexterous control.

## Figures and Tables

**Table 1:** Hardware, sensing, and control parameters directly relevant to the physical piano experiments.

Component	Specifications Used in This Study
Piano	Yamaha P-48B; 88 keys; white-key width approximately 22–23 mm; black-key width approximately 10–13 mm; white-key travel approximately 9–11 mm; MIDI onset, key number, and velocity acquired through USB TO HOST.
Dexterous hand	Beyond Hand end-effector; the policy controls 16 finger joint coordinates and 2 wrist coordinates; joint-position commands are tracked by embedded position servos using joint-angle feedback. Detailed hand transmission and mechanical design are reported in Ref. [21].
Global hand positioning	UR5 collaborative arm; the policy action additionally contains 3 translational coordinates for global forearm/hand positioning over the keyboard.
Control and sensing rates	High-level policy/control trajectory at 40 Hz; exported commands interpolated to 100 Hz for hardware playback; low-level joint servos use real-time joint-angle feedback and PD position control.
Physical evaluation	One complete physical execution for each 30 s excerpt; MIDI and joint-angle trajectories recorded during the reported execution.

**Table 2:** Principal SAC and training hyperparameters used in the reported experiments.

Parameter	Stage 1	Stage 2
Actor learning rate	3 × 10^−4^	1 × 10^−4^
Critic learning rate	1 × 10^−3^	1 × 10^−3^
Entropy/alpha learning rate	1 × 10^−3^	1 × 10^−3^
Discount factor γ	0.85	0.90
Polyak coefficient τ	0.005	0.005
Batch size	4096	4096
Replay capacity	1024 vector slots (262,144 transitions with 256 environments)	Same
Random-action warm-up	300 vector steps	0
Learning starts	1000 vector steps	1000 vector steps
Update schedule	Every outer step after learning starts	Same
Gradient iterations per update	32	32
Target entropy	−0.5 × action dimension = −10.5	Same
Initial entropy coefficient α	0.2	0.2
Gradient clipping	Disabled (grad_norm_clip = 0.0)	Disabled (grad_norm_clip = 0.0)

**Table 3:** Stage-dependent RewardManager weights used in the reported experiments.

Reward/Penalty	Stage 1	Stage 2 Canon	Stage 2 Für Elise/Beethoven
Key-press reward	1.0	2.0	2.0
Fingering reward	1.0	1.5	1.5
Wrong-key penalty	Embedded in key-press term; no independent weight	Same	Same
Forearm power	0.2	4.6	4.0
Wrist power	0.176	0.1	0.1
Finger power	0.2	1.0	1.0
Forearm velocity	0.2	2.6	2.2
Wrist velocity	0.176	0.1	0.1
Finger velocity	0.2	1.0	1.0
Forearm acceleration	0.2	6.4	5.5
Wrist acceleration	0.176	0.1	0.1
Finger acceleration	0.2	1.0	1.0
Finger-speed limit	Not enabled	0.4; vmax = 6.5	0.3; vmax = 6.5
Collision penalty	1.0	1.0	1.0
Natural posture/stretch	1.0	1.0	1.0
Wrist-pitch term	1.0	1.0	1.0
Forward-press constraint	1.0	1.0	1.0

Note: The weights above are the external RewardManager weights. The power, velocity, acceleration, and finger-speed terms also include the internal coefficients defined in Equations (15)–(18); Stage 2 additionally applies the implemented shaping function to these regularization terms.

## Data Availability

The original contributions presented in this study are included in the article and its Supplementary Materials. Further inquiries can be directed to the corresponding author.

## Supplementary Materials

The following supporting information can be downloaded at: https://www.mdpi.com/article/10.3390/biomimetics11090610/s1. Video S1: Robotic piano-playing demonstrations. The video is divided into three parts, sequentially demonstrating the simulation and physical (real-world) executions for the three musical excerpts: Part 1—Für Elise, Part 2—Canon, and Part 3—Beethoven’s Symphony No. 5.
